# *E*-Test or Agar Dilution for Metronidazole Susceptibility Testing of *Helicobacter pylori*: Importance of the Prevalence of Metronidazole Resistance

**DOI:** 10.3389/fmicb.2022.801537

**Published:** 2022-03-14

**Authors:** Jinnan Chen, Yu Huang, Zhaohui Ding, Xiao Liang, Hong Lu

**Affiliations:** Division of Gastroenterology and Hepatology, Key Laboratory of Gastroenterology and Hepatology, Ministry of Health, School of Medicine, Renji Hospital, Shanghai Institute of Digestive Disease, Shanghai Jiao Tong University, Shanghai, China

**Keywords:** *Helicobacter pylori*, susceptibility, agar dilution, *E*-test, metronidazole

## Abstract

**Background:**

A number of studies have shown that *E*-test overestimated the presence of *Helicobacter pylori* resistance compared to agar dilution.

**Objective:**

The purpose of this study was to explore whether *E*-test could be an alternative for agar dilution to detect the metronidazole susceptibility of *H. pylori*.

**Method:**

*E*-test and agar dilution were used to assess the susceptibility of *H. pylori* to metronidazole, clarithromycin, and levofloxacin in 281 clinical isolates obtained from China where the resistance was high. Cohen’s kappa analysis, McNemar’s test, and essential and categorical agreement analysis were performed for these two methods.

**Results:**

Overall, the result of the *E*-test showed a similar prevalence of resistance rate to all antibiotics compared with agar dilution. The essential agreement of the *E*-test method and agar dilution in the evaluation susceptibility of *H. pylori* to clarithromycin and levofloxacin was moderate at 89.0 and 79.7%, respectively, but only 45.9% for metronidazole. The results shown by a categorical agreement (CA) between the *E*-test and agar dilution were 100% for both clarithromycin and levofloxacin. As for metronidazole, the CA was 98.7%, no major error was identified, and the rate of a very major error was 1.8%.

**Conclusion:**

*E*-test can be an alternative method to detect the metronidazole susceptibility of *H. pylori*.

## Introduction

All successful infectious disease therapies are directly or indirectly based on susceptibility. For regions where resistance is common, susceptibility-guided tailored treatment is typically required to achieve high cure rates. This is especially true with *Helicobacter pylori* infections (Rimbara et al., 2011; Sugano et al., 2015). The worldwide increase in antibiotic-resistant *H. pylori* has resulted in relatively poor cure rates with empiric therapy (Graham and El-Serag, 2021). However, even in regions with only modest levels of resistance, antibiotic susceptibility testing prior to *H. pylori* treatment has been shown to increase the eradication rate compared to empirical treatment (Wenzhen et al., 2010; Lopez-Gongora et al., 2015). Metronidazole, clarithromycin, and levofloxacin are among the most commonly used antibiotics in the clinical treatment of *H. pylori*, and the need to assess bacterial antibiotic susceptibility patterns before treatment has received increasing attention (Dang and Graham, 2017).

At present, there are four methods widely used for traditional microbial susceptibility testing, including agar dilution, *E*-test, disk diffusion, and broth microdilution. Agar dilution is believed to be the gold standard for *H. pylori* susceptibility testing, although this method is time-consuming and laborious (Valdivieso-Garcia et al., 2009). Broth microdilution is rarely used in the detection of drug susceptibility to *H. pylori* because some studies suggest that it is difficult for it to grow in liquid (DeCross et al., 1993; Xia et al., 1994).

*E*-test, as an alternative method, combines the principle of dilution and diffusion methods by placing a single strip containing an increased antibiotic concentration on the surface of the agar medium and reading the intersection of the bacterial growth zone and the inhibition zone to determine the minimum inhibitory concentration (MIC). At present, due to the convenience of the implementation of *E*-test, it is widely used in the clinical microbiological laboratory. However, some studies reported that it cannot be used for evaluating the metronidazole susceptibility of *H. pylori* because of its inconsistency with agar dilution (von Recklinghausen and Ansorg, 1995; Alarcon et al., 1998; Osato et al., 2001b).

The purpose of this study was to perform the susceptibility tests by agar dilution method and *E*-test method to verify whether *E*-test could be an alternative way for detecting antibiotic susceptibility, especially metronidazole.

## Materials and Methods

### Study Population

From July 2019 to December 2020, a total of 281 *H. pylori* isolates were obtained from patients who underwent endoscopy at Renji Hospital, School of Medicine, Shanghai Jiao Tong University, China.

### *Helicobacter pylori* Strains

During endoscopy, two biopsies were collected from the antrum of the stomach and cultured on brain heart infusion (BHI) agar medium (Oxoid, Stoke, Basin, United Kingdom) containing 5% defibrinated sheep blood, 5 mg/L trimethoprim, 10 mg/L vancomycin, 20 U/L polymyxin B, and 10 mg/L nalidixic acid under microaerophilic conditions (85% N_2_, 10% CO_2_, and 5% O_2_) at 37°C. The strains were confirmed according to Gram-negative, positive urease, oxidase, and catalase reaction, and its morphology was spiral or curved. The strains were collected in BHI broth with glycerol at 4°C and stored at −80°C. Before the susceptibility test, bacteria were resuscitated and subcultured on BHI agar medium (Oxoid, Stoke, Basin, United Kingdom). ATCC43504 was used as the quality control with MIC from 64 to 256 mg/L for metronidazole, from 0.016 to 0.125 mg/L for clarithromycin, and from 0.032 to 0.125 mg/L for levofloxacin.

### Agar Dilution Method

Agar dilution was performed based on the protocol presented by the Clinical Laboratory Standards Institute. In brief, metronidazole, clarithromycin, and levofloxacin were dissolved in dimethyl sulfoxide. The drug was added to the agar medium to produce continuous twofold dilutions with concentrations ranging from 0.032 to 256 mg/L for metronidazole, from 0.032 to 256 mg/L for clarithromycin, and from 0.032 to 32 mg/L for levofloxacin. Bacterial suspensions (0.5 McFarland) were prepared with sterile saline. The adjusted inoculum (2–5 ul) was then delivered to each plate by an inoculator (Sakuma Seisaku, Tokyo, Japan). After 3 days of incubating the plates in a microaerobic environment, the lowest concentration of the drug that prevented the visible growth of a bacterium (excluding single colony or multiple tiny colonies) was defined as the MIC. The clarithromycin and levofloxacin susceptibility tests were used as positive references to confirm the reliability of our methodology.

### Broth Microdilution Method

Twofold dilutions of metronidazole, clarithromycin, and levofloxacin dissolved in BHI containing 5% fetal calf serum were added to 96 wells with concentrations ranging from 0.032 to 256 mg/L, 0.032 to 256 mg/L, and 0.032 to 32 mg/L, respectively. For the *H. pylori* preparation, 1 ml of sterile saline was adjusted to 0.5 McFarland, and *H. pylori* was inoculated to each well at 5 × 10^5^ colony-forming units. The growth was examined after incubating the plates in a microaerobic environment for 5–7 days (DeCross et al., 1993).

### *E*-Test Method

One hundred microliters of *H. pylori* suspension (3 McFarland) was inoculated onto the agar plate without antibiotics. After allowing to stand for a few minutes, *E*-test strip was then placed on the center of the agar plate. The plates were then incubated under a microaerobic environment for 72 h. The endpoint of the *E*-test was read as the interception of the graded strips with the elliptical zone of inhibition. If the endpoints were not within the twofold dilution range, it would be rounded up to the next highest twofold dilution for MIC assessment.

### Discrepancy Analysis

Isolates with an inconsistent interpretation of susceptibility after initial testing by agar dilution and *E*-test were further tested for four additional times. The most frequent results of agar dilution were considered as the MIC reference value of the isolates, and the most frequent results of the *E*-test were used as the final MIC value of the isolates tested by it.

### Statistical Analysis

The isolates were classified as resistant based on the breakpoint for each drug as established by EUCAST (MIC ≥ 8 mg/L for metronidazole, MIC ≥ 0.5 mg/L for clarithromycin, and MIC ≥ 1 mg/L for levofloxacin). The disagreement of two tests was performed by McNemar’s test and Cohen’s kappa analysis. Agar dilution method and broth microdilution method were used as the reference methods and compared with *E*-test separately. Essential agreement (EA) was determined by calculating the percentage of isolates whose MIC produced by *E*-test were within ± 1 doubling dilution of that produced by the reference method. Categorical agreement (CA) was determined by calculating the percentage of isolates that occupied the same susceptibility category as tested by the reference method and *E*-test. A very major error (VME) was defined as isolates being resistant by the reference method and susceptible by the *E*-test. Major error (ME) was defined as an isolate being susceptible by the reference method and resistant by the *E*-test.

## Results

### Clinical Characteristics

Table 1 shows the clinical information of strains. The median age of the hosts was 46 years (16–73), with 101 (35.9%) being male individuals and 180 (64.1%) being female individuals.

**TABLE 1:** Clinical information of the patients.

Clinical information	
**Age**	
Median	46 (16–73)
**Gender**	
Male	101 (35.9)
Female	180 (64.1)
**Endoscopic diagnosis**	
Chronic superficial gastritis	119 (42.3)
Chronic gastritis	120 (42.7)
Duodenal ulcer	25 (8.9)
Gastric ulcer	10 (3.6)
Complex ulcer	7 (2.5)
**Previous treatment**	
No treatment history	23 (8.2)
One failure	147 (52.3)
Two or more failures	111 (39.5)

### Agreement of Susceptibility Results

Table 2 shows that the resistance rates of the isolated *H. pylori* to metronidazole, clarithromycin, and levofloxacin, when assessed in terms of a binary outcome (susceptible/resistant), were 71.5, 88.6, and 80.4%, respectively, as tested by the agar dilution method. As for clarithromycin and levofloxacin, the *E*-test showed same susceptibility pattern (McNemar’s test, *P* = 1.00). For metronidazole, the *E*-test showed a slight difference (McNemar’s test, *P* = 0.062) when compared with the agar dilution method. Cohen’s kappa analysis was further performed to determine the consistency and accuracy of the *E*-test (Table 2) as the agar dilution method was used as the reference in this study. The kappa values indicated a substantial agreement for metronidazole (0.96; 95% CI: 0.92–1.00), clarithromycin (1.00; 95% CI: 1.00–1.00), and levofloxacin (1.00; 95% CI: 1.00–1.00) between the *E*-test and the agar dilution method.

**TABLE 2:** Resistance rates based on the agar dilution method and *E*-test.

Antibiotic	Clinical break point (mg/L)	MIC_50_ (mg/L)		MIC_90_ (mg/L)		Resistance rate (%)		McNemar’s test (*P*-value)	Kappa coefficient (95% CI)
		Agar dilution	*E*-test	Agar dilution	*E*-test	Agar dilution	*E*-test		
Metronidazole	8	32	≥256	128	≥256	201/281 (71.5)	206/281 (73.3)	0.062	0.96 (0.92–1.00)
Clarithromycin	0.5	64	128	128	≥256	248/281 (88.3)	248/281 (88.3)	1.000	1.00 (1.00–1.00)
Levofloxacin	1	16	≥32	≥32	≥32	226/281 (80.4)	226/281 (80.4)	1.000	1.00 (1.00–1.00)

As for the five strains with inconsistent interpretations, four replicated tests were performed. The results showed that the MIC of two strains was 8 ug/ml and the remaining strains were less than 2 ug/ml when interpreted by the agar dilution method. However, based on the *E*-test, all five strains were considered as resistant strains (Supplementary Table 1).

Supplementary Table 2 shows the antimicrobial susceptibility test results of *H. pylori* when tested by broth microdilution. Cohen’s kappa analysis showed a moderate agreement for metronidazole (0.47; 95% CI: 0.35–0.59), clarithromycin (0.49; 95% CI: 0.33–0.64), and levofloxacin (0.59; 95% CI: 0.47–0.71) when compared with the *E*-test. Similar results can also be detected between the agar dilution method and the broth microdilution.

### Essential and Categorical Agreement

**FIGURE 1:**
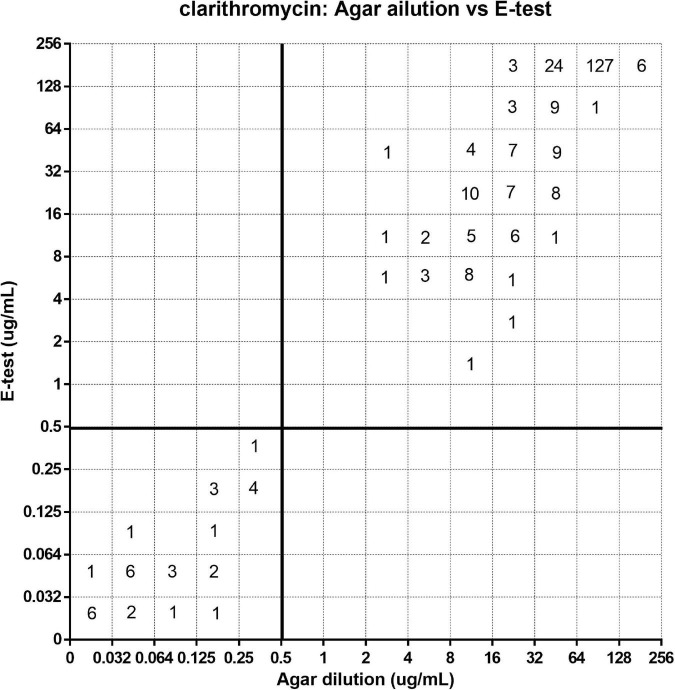
Error rate-bounded analysis of clarithromycin’s minimum inhibitory concentration (MIC) of 281 strains tested by agar dilution and *E*-test.

**FIGURE 2:**
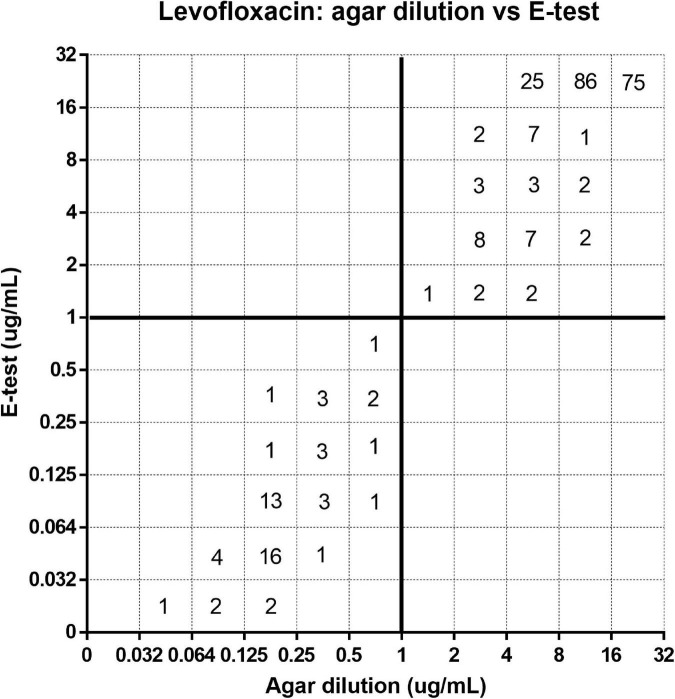
Error rate-bounded analysis of levofloxacin’s minimum inhibitory concentration (MIC) of 281 strains tested by agar dilution and *E*-test.

**FIGURE 3:**
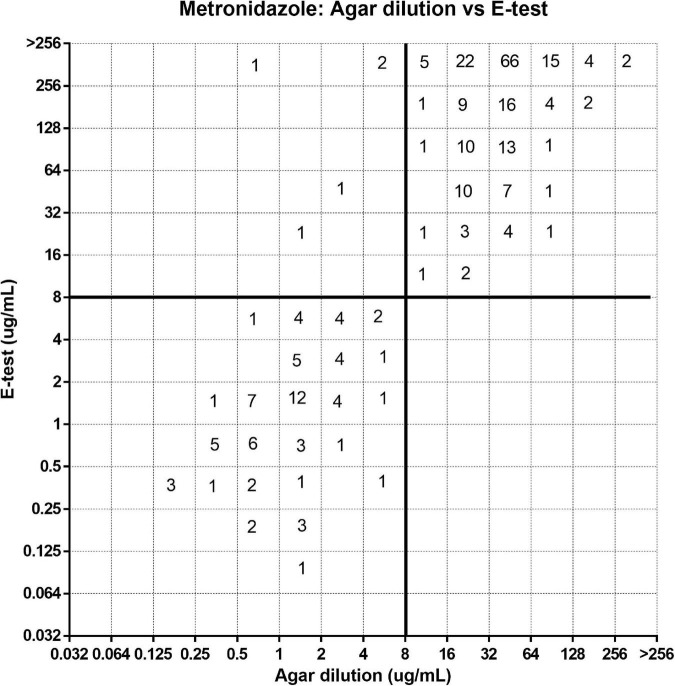
Error rate-bounded analysis of metronidazole’s minimum inhibitory concentration (MIC) of 281 strains tested by agar dilution and *E*-test.

Figures 1–3 show the distribution of MIC for the agar dilution and the *E*-test. The EA for these two methods (Table 3) indicated a moderate correlation for testing the susceptibility of clarithromycin (84%) and levofloxacin (79.7%). However, for metronidazole, the EA between these two methods was low (45.9%). On the contrary, the CA was high (>98%) for all three antibiotics’ susceptibility test comparison without VME; only 1.8% of the strains tested for metronidazole was observed as ME between the *E*-test and agar dilution.

**TABLE 3:** Essential and categorical agreement of the agar dilution and *E*-test.

Antibiotic	% Essential agreement (*n*)	% Categorical agreement (*n*)	% ME (*n*)	% VME (*n*)
Metronidazole	129/281 (45.9)	276/281 (98.2)	5/281 (1.8)	0
Clarithromycin	250/281 (89.0)	281/281 (100)	0	0
Levofloxacin	224/281 (79.7)	281/281 (100)	0	0

*VME, very major errors [the minimum inhibitory concentration (MIC) of the drug was interpreted as resistance by agar dilution but sensitive by the E-test]; ME, major errors (the MIC of the drug was interpreted as sensitive by agar dilution but resistance by the E-test).*

Supplementary Table 3 shows the EA and CA between broth dilution and *E*-test. The EA for these two methods demonstrated a moderate agreement for metronidazole (45.2%), clarithromycin (65.8), and levofloxacin (67.3%). The results of the CA showed a substantial agreement (>80%) for all three antibiotics between broth dilution and *E*-test. Moreover, compared with the broth microdilution method, when the *E*-test was used to detect the drug susceptibility of metronidazole, clarithromycin, and levofloxacin, the probability of ME occurrence was 6.8, 6.4, and 7.1%, respectively. Besides this, the probability of VME occurrence was 12.4, 4.6, and 6.0%, respectively.

## Discussion

Accurate knowledge of the resistance pattern can effectively improve the success rate of the treatment, avoid the use of unnecessary antibiotics, and improve the compliance (Neri et al., 2003; Zhou et al., 2016). Agar dilution is regarded as the gold standard for bacterial susceptibility tests, although it is cumbersome and time consuming. *E*-test is often used as a substitution in clinical practice because of its convenience in detecting single or few isolates. However, the results with *E*-test are more difficult to interpret (Perna, 2003).

Previous studies have compared the efficacy of the above-mentioned two methods and found that the EA and CA of the *E*-test and agar dilution were both in high agreement for amoxicillin, clarithromycin, and levofloxacin (Wang et al., 2000; Cheng et al., 2015; Miftahussurur et al., 2020). However, for metronidazole, a class of nitroimidazole compounds, the EA of the *E*-test and agar dilutions is generally considered to be low, which is often less than 60% in previous studies, and the CA remains controversial as some laboratories have reported that about 5–32% of bacteria exhibited a change in the pattern of metronidazole resistance between these two methods (Alarcon et al., 1998; Chaves et al., 1999; Wang et al., 2000; Osato et al., 2001a,b; Perna, 2003; Ogata et al., 2014; Miftahussurur et al., 2020). Understanding the drug susceptibility pattern of metronidazole is of great significance for the treatment of *H. pylori*, although it has been reported that metronidazole resistance *in vitro* does not necessarily indicate failure of treatment. In practice, a high dose of metronidazole can overcome drug resistance, but its side effects are large and patient compliance is poor. Besides this, in our past study, we found that the metronidazole-containing therapy had the highest eradication rate when administered under the guidance of drug susceptibility (Chen et al., 2019; Luo et al., 2020).

China is a country with a high antibiotic resistance rate of *H. pylori*. In our study, the drug resistance rates of metronidazole, levofloxacin, and clarithromycin were 71.5, 80.4, and 88.3%, respectively, which were higher than the primary antibiotic resistance rates in China (Hu et al., 2017). There may have been some selection bias in our study because this was not a clinical trial, and a large proportion of the included strains was isolated from patients who had a failed *H. pylori* treatment. In our study, the results showed that the EA between the *E*-test and the agar dilution method for the metronidazole susceptibility test was poor (45.9%), but the categorical agreement was high (98.2%). Differently from some previous studies (Cederbrant et al., 1993; Alarcon et al., 1998; Ogata et al., 2014), VME was not found in our work for testing metronidazole, and for five strains with changes in susceptibility pattern, all were metronidazole-susceptible strains as identified by agar dilution, which were regarded as resistant by the *E*-test. At the same time, unlike clarithromycin and levofloxacin, the two methods showed obvious discrepancies in the MIC distribution for metronidazole. The values obtained by the *E*-test were often 2–5 times higher than those by agar dilution, especially when the MIC is higher than the clinical breakpoint. This is consistent with some previous reports (Citron et al., 1991; Glupczynski et al., 2002; Perna, 2003). Thus, the percentage of cases overestimated by the *E*-test depends on the relative proportion with infections deemed resistant by agar dilution. We also analyzed the consistency of the broth microdilution method and the *E*-test method, and the results indicated that the EA between them was poor (45.2%). Meanwhile, the CA was 80.8%, lower than clarithromycin (89.0%) and levofloxacin (86.9%), accompanied by 12.4% VME and 6.8% ME. There are few studies on whether the broth dilution method can be used to test the metronidazole susceptibility of *H. pylori*. Charles found 12.3% ME in broth dilution and *E*-test, while in Francesca’s study, there was no significant difference between the results of agar dilution using RPMI 1640 medium and broth microdilution (Hachem et al., 1996; Sisto et al., 2009). In our study, there was a certain difference in CA between the agar dilution method and the broth microdilution method. If the agar dilution method was used as the reference method, the probability of ME and VME of the broth dilution method was about 11, 13.1, and 19.2% for clarithromycin, levofloxacin, and metronidazole, respectively (data not shown), but there was no significant difference with the *E*-test. We think that this may be due to the phenomenon of induced drug resistance in the culture process, but more studies are needed to further confirm this.

Four replicates were performed on these five strains with MEs, and the results can be divided into two categories for discussion. For the first category, after the MIC of bacteria was identified by the agar dilution method, its value was around the breakpoint of metronidazole. As mentioned earlier, due to the value of the *E*-test being usually higher than that of the agar dilution method, it may be difficult to determine accurately for such bacteria with a MIC near the breakpoint. For the second category, these bacteria might have mixed infection. When MIC was determined by agar dilution, the bacterium was considered sensitive according to the protocol, even if monoclone or tiny clones were present on the culture plate. However, in the *E*-test, the inoculation amount of bacteria was larger than in agar dilution, and there were still many scattered clones in the inhibition zone on the culture plate. Thus, we determined that it was drug-resistant bacteria interpreted by *E*-test, leading to the discordance with agar dilution. Such heterogeneity made us realize that, when testing for the metronidazole susceptibility of *H. pylori* by agar dilution, the susceptibility of the bacterium can be controversial if monoclone or tiny clones are present on the plate. However, we cannot accurately assess the susceptibility of these bacteria unless the corresponding clinical outcome is supported.

The MIC discrepancy between the agar dilution and the *E*-test is related, in part, to the details of placing the strips on the plates and the experience in the interpretation of the results (Citron et al., 1991; Cederbrant et al., 1992). In the procedure of preparing the agar dilution medium, variables including drug degradation, drug weighing error, and heterogeneous mixing of the drug and medium may influence the antibiotic activities. As for the *E*-test, ambient temperature, humidity, and depth of medium, which may affect the diffusion efficiency of the drug on the medium, may have an effect on the results. In addition, cryopreservation, the continuous subculture of bacteria before the experiment, and the motility of the bacteria may also interfere with the experimental results (Dore et al., 1999; Han et al., 1999). Meanwhile, metronidazole itself is also affected by the oxygen concentration. Chida-Sakata et al. reported that some bacteria whose MICs is overestimated by the *E*-test had a MIC concordance to the agar dilution after 24 h of pre-incubation in an anaerobic environment; however, this phenomenon remains unexplained (Cederbrant et al., 1992; Chida-Sakata et al., 1999). Considering that the *E*-test has only a few discordances with the agar dilution in determining the susceptibility pattern of metronidazole, it is suggested that, when the *E*-test is used for metronidazole susceptibility testing, the prevalence of resistance strains may be overestimated, leading to a decrease in the effective use of metronidazole as well as an overestimation of the ability of a regimen to overcome metronidazole resistance. However, this is less of an issue in areas where metronidazole resistance is widespread, and the drug would be commonly used for susceptible strains.

## Conclusion

In general, although discrepancies between the *E*-test and the agar dilution method for determining the susceptibility rates in *H. pylori* are observed for metronidazole more often than for clarithromycin and levofloxacin, these discrepancies are trivial in areas with high-level metronidazole resistance. Using the *E*-test is an acceptable alternative method when this leads to few missed treatment opportunities, as very few isolates would have been classified as susceptible to metronidazole by the agar dilution method, rather than to risk being misclassified as resistant by the *E*-test.

## Data Availability Statement

The raw data supporting the conclusions of this article will be made available by the authors, without undue reservation.

## Ethics Statement

This study conformed with the Ethical Guidelines of the World Medical Association Declaration of Helsinki, Ethical Principles for Medical Research Involving Human Subjects. The study was approved by the Ethics Committee of Renji Hospital, Shanghai Jiao Tong University School of Medicine.

## Author Contributions

HL contributed to study concept and design, carried out important and critical revision of the manuscript, and obtained funding. JC, YH, and ZD contributed to the acquisition of data. JC and YH contributed to the analysis and interpretation of data. JC and ZD contributed to the drafting of the manuscript. JC performed the statistical analysis. All authors contributed to the article and approved the submitted version.

## Conflict of Interest

The authors declare that the research was conducted in the absence of any commercial or financial relationships that could be construed as a potential conflict of interest.

## Publisher’s Note

All claims expressed in this article are solely those of the authors and do not necessarily represent those of their affiliated organizations, or those of the publisher, the editors and the reviewers. Any product that may be evaluated in this article, or claim that may be made by its manufacturer, is not guaranteed or endorsed by the publisher.
