# Supplementary material for: E-Test or Agar Dilution for Metronidazole Susceptibility Testing of Helicobacter pylori: Importance of the Prevalence of Metronidazole Resistance
Source: Front Microbiol. 2022 Mar 14;13:801537. doi: 10.3389/fmicb.2022.801537 (PMC8964178; doi:10.3389/fmicb.2022.801537)
Supplement: Supplementary file 1 [file Data_Sheet_1.doc]

Supplementary table 1 List of final results for isolates of *H. pylori* (n=5) that following discrepancy resolution adjudication

| Isolates no. | Agar dilution | | Broth microdilution | | E-test | |  |
| --- | --- | --- | --- | --- | --- | --- | --- |
|  | MIC  μg/ml | EUCAST MIC interpretation | MIC  μg/ml | EUCAST MIC interpretation | MIC  μg/ml | EUCAST MIC interpretation | Error (agar versus E-test) |
| 6026 | 8 | sensitive | 256 | resistance | 256 | resistance | Major error |
| 6073 | 8 | sensitive | 256 | resistance | 256 | resistance | Major error |
| 6071 | 2 | sensitive | 256 | resistance | 256 | resistance | Major error |
| 6097 | 1 | sensitive | 256 | resistance | 64 | resistance | Major error |
| 7165 | 1 | sensitive | 1 | sensitive | 256 | resistance | Major error |

**Table 2 resistance rates based on broth microdilution dilution**

| Antibiotic | Clinical break point (mg/L) | MIC50 (mg/L) | MIC90 (mg/L) | Resistance rate (%) | Mcnemar’s test (P-value) | | Kappa Coefficient (95% CI) | |
| --- | --- | --- | --- | --- | --- | --- | --- | --- |
|  |  | BD | BD | BD | AD vs BD | E-test vs BD | AD vs BD | E-test vs BD |
| Metronidazole | 8 | 64 | 128 | 222/281 (79.0) | 0.008 | 0.04 | 0.46 (0.34-0.58) | 0.47 (0.35-0.59) |
| Clarithromycin | 0.5 | 128 | ≥256 | 244/281 (86.8) | 0.47 | 0.47 | 0.49 (0.33-0.64) | 0.49 (0.33-0.64) |
| Levofloxacin | 1 | 16 | ≥32 | 223/281(79.4) | 0.74 | 0.74 | 0.59 (0.47-0.71) | 0.59 (0.47-0.71) |

AD: agar dilution

BD: broth microdilution

**Table 3 essential and categorical agreement of broth microdilution and E-test**

| Antibiotic | % Essential  agreement (n) | % Categorical agreement (n) | % ME  (n) | % VME  (n) |
| --- | --- | --- | --- | --- |
| Metronidazole | 127/281 (45.2) | 227/281 (80.8) | 19/281 (6.8) | 35/281 (12.4) |
| Clarithromycin | 185/281 (65.8) | 250/281 (89.0) | 18/281 (6.4) | 13/281 (4.6) |
| Levofloxacin | 189/281 (67.3) | 244/281 (86.9) | 20/281 (7.1) | 17/281 (6.0) |

VME: very major errors, MIC of drug was interpreted as resistance by broth microdilution but sensitive by E-test.

ME: major errors, MIC of drug was interpreted as sensitive by broth microdiution but resistance by E-test.
